# Enhancing pharmacists’ role in developing countries to overcome the challenge of antimicrobial resistance: a narrative review

**DOI:** 10.1186/s13756-018-0351-z

**Published:** 2018-05-02

**Authors:** M. H. F. Sakeena, Alexandra A. Bennett, Andrew J. McLachlan

**Affiliations:** 1 Department of Pharmacy, Faculty of Allied Health Sciences, University of Peradeniya, Peradeniya, Sri Lanka; 2 Sydney Pharmacy School, The University of Sydney, Sydney, NSW Australia; 3 NSW Therapeutic Advisory Group, Sydney, NSW Australia

**Keywords:** Antibiotics, Antimicrobial resistance, Pharmacists, Pharmacy education, Pharmacy services, Developing country

## Abstract

**Background:**

Antimicrobial resistance (AMR) is a global health challenge and developing countries are more vulnerable to the adverse health impacts of AMR. Health care workers including pharmacists can play a key role to support the appropriate use of antimicrobials in developing countries and reduce AMR.

**Objective:**

The aim of this review is to investigate the role of pharmacists in the appropriate use of antibiotics and to identify how the pharmacists’ role can be enhanced to combat AMR in developing countries.

**Method:**

The databases MEDLINE, EMBASE, Web of Science and Google Scholar were searched for articles published between 2000 and the end of August 2017 that involved studies on the role of pharmacists in developing countries, the expanded services of pharmacists in patient care in developed countries and pharmacists’ contributions in antimicrobial use in both developed and developing nations.

**Key findings:**

In developing countries pharmacists role in patient care are relatively limited. However, in developed nations, the pharmacists’ role has expanded to provide multifaceted services in patient care resulting in improved health outcomes from clinical services and reduced health care costs. Success stories of pharmacist-led programs in combating AMR demonstrates that appropriately trained pharmacists can be part of the solution to overcome the global challenge of AMR. Pharmacists can provide education to patients enabling them to use antibiotics appropriately. They can also provide guidance to their healthcare colleagues on appropriate antibiotic prescribing.

**Conclusions:**

This review highlights that appropriately trained pharmacists integrated into the health care system can make a significant impact in minimising inappropriate antibiotic use in developing countries. Strengthening and enhancing the pharmacists’ role in developing countries has the potential to positively impact the global issue of AMR.

## Background

Antimicrobial resistance (AMR) is a serious global health challenge [1]. It is predicted that by 2050 there will be more than ten million deaths per year attributed to AMR [2]. Further, it is predicted that the greatest number of these deaths will be in developing countries [2]. Therefore, there is an urgent need to take action to minimize the emergence of antimicrobial resistant bacteria in developing countries [3, 4]. The management of development and spread of AMR requires a multifaceted approach, including the participation of all healthcare workers [5]. According to the first objective of the World Health Organization (WHO) global action plan on AMR, avoiding overuse and misuse of antibiotics requires healthcare professionals awareness and understanding of AMR with effective communication, education and training [6]. In this context, healthcare professionals have a key role to play to optimize the use of antibiotics in the community.

Pharmacists are important members of the healthcare team and they play a major role in medicine use and the provision of advice regarding appropriate medicines use [7]. Education and training of pharmacists has the potential to influence the behaviour of healthcare team members and consumers [8] as part of a multidimensional strategy for changing practice and ensure the quality use of antibiotics [9]. They are well placed to improve the understanding of antibiotics and inform their judicious use by direct contact with consumers in the community [10] and in hospital [11]. Consumer education is an important component of the fight against AMR and pharmacists can improve consumer’s awareness of safe and appropriate medication practices concerning antibiotics [12]. Comprehensive and relevant education and training on the use of antibiotics and AMR is essential for pharmacists in order that they may take a leading role in changing behaviours regarding antibiotic consumption in all healthcare settings.

Numerous studies have highlighted that inappropriate medication practices are relatively common in the community and hospital settings of developing countries [13–15]. For example, use of antibiotics without medical prescriptions is a well described practice, despite regulation prohibiting the supply of antibiotics in this manner [16–18]. Absence of qualified pharmacists is one factor that has contributed to these inappropriate practices in community pharmacies [19]. Similarly, overprescribing of antibiotics in hospitals in developing countries is common, and the lack of clinical pharmacists in hospitals in developing countries may be a contributing factor [20].

Several reviews have addressed and proposed possible solutions to the challenge of AMR in developing countries [21, 22]. However, there are currently relatively few reports on how pharmacists can play a role in overcoming the challenge of AMR in developing countries. In order to ensure the recommended multidisciplinary approaches taken (WHO), strategies that reduce AMR in developing countries, should consider the contribution of appropriately qualified and trained pharmacists. Therefore, the aim of this narrative review is to investigate how the pharmacists’ role can be further enhanced to provide judicious and appropriate antibiotic use in developing countries.

## Method

### Literature search strategy

A literature search was conducted to identify articles, published between 2000 and the end of August 2017 that involved studies on antimicrobial use or AMR involving pharmacists in community and hospital settings of developing countries using databases such as EMBASE, MEDLINE and Web of Science.

The following ‘Medical Subject Headings’ (MeSH) terms were used to search articles: (Antibiotics.mp.) OR (Anti-Bacterial Agents) OR (Antimicrobial agents.mp. or Anti-Infective Agents) OR (Drug Resistance, Bacterial or Antimicrobial resistance.mp. or Drug Resistance, Microbial) AND (Community pharmacy.mp. or Pharmacies) OR (Community Pharmacy Services/ or Pharmacists/ or community pharmacist.mp.) OR (Professional Role/ or Pharmacy Service, Hospital/ or Pharmacy/ or Pharmacists/ or Pharmacy practice.mp. or Pharmaceutical Services) OR (Clinical pharmacy.mp.) AND (Pharmacist OR Community pharmacy OR Community pharmacist OR Clinical Pharmacy OR Clinical pharmacist OR Pharmaceutical care) AND (Attitude OR Perception OR Barriers).

The following keywords were used to search articles in Web of Science: (“Antibiotics” OR “Antimicrobials”) AND (“Community pharmacy” OR “Community pharmacist”) AND (“Hospital pharmacist” OR “Clinical pharmacist”). Google Scholar was also used to search for articles with the appropriate keywords. Cross-references of articles identified using these databases were also searched. National pharmacy journals of developing countries were also searched for relevant articles.

### Selection of articles

This review focuses on three main areas; firstly, the current role of pharmacists in developing countries; secondly, pharmacists’ services in patient care in developed countries; and, thirdly, success stories involving pharmacists that optimised antibiotic use and reduced AMR in both developed and developing nations. Articles were scrutinised to identify those that addressed the above. Only articles and abstracts published in English were included in the review. This will not limit this review, because authors have searched different national journals from developing countries which publishes articles in English and available online.

## Results

### Pharmacists’ roles – Current scenario in developing countries

The challenges and barriers related to pharmacists’ roles in pharmaceutical care in developing countries identified from recently published research articles are presented in Table 1. These studies are based on pharmacists’ reports from a variety of developing countries including those in Asia [23–26], Africa [27, 28], South America [29] and the Middle-East [30–32]. These results highlight that challenges exist in both community [23–26, 29, 30] and hospital [27, 28, 31] provision of pharmaceutical care services. The major barriers to the delivery of comprehensive pharmacy services include the shortage of pharmacists, the lack of pharmaceutical care training programs and institutional obstacles.

**Table 1:** Pharmacists perspectives about challenges and barriers related to pharmaceutical care services in developing countries

Authors	Country	Setting	Study design	Participants	Objectives of the study	Outcomes of the study
Yu Fang et al., 2011 [23]	China	Community	Questionnaire based survey	Pharmacists	To explore the perceptions of community pharmacists towards the concept of pharmaceutical care, and barriers to implementation of pharmaceutical care in China	Lack of external conditions for developing or providing pharmaceutical care, lack of time and skills, absence of information and economic incentive, and lack of full support from other health professionals.
Hashmi et al., 2017 [24]	Pakistan	Community	Semi-structured interview - qualitative	Pharmacists	To explore knowledge, perception and attitude of community pharmacists about extended pharmaceutical services in the city of Lahore, Pakistan	Lack of personal knowledge, poor public awareness, inadequate physician-pharmacist collaboration and deprived salary structures
Kho et al., 2017 [26]	Malaysia	Community	Semi-structured interview - qualitative	Pharmacists	To obtain in-depth information on the provision of professional pharmacy services by community pharmacies in Sarawak, Malaysia	Lack of time, personnel and physical space constrained services provision, cultural issues, lack of committed customers, stock availability and price issues
Sancar et al., 2013 [25]	Turkey	Community	Questionnaire based survey	Pharmacists	To assess Turkish community pharmacists’ points of view about pharmaceutical care practice in Turkey	Lack of knowledge of drugs and disease states, lack of technical knowledge of how to provide pharmaceutical care practice, lack of communication with physicians and stationary workload
Bilal et al., 2017 [27]	Ethiopia	Hospital	Cross-sectional survey	Pharmacists	To assess the status, challenges and way forward of clinical pharmacy services in Ethiopia	Shortage of staff, lack of awareness, lack of support from management, hospital setup, incentives, and gaps in the curriculum
Salim et al., 2016 [28]	Sudan	Hospital	Exploratory corss-sectional study	Pharmacists	To explore the self-perception of clinical pharmacists of their impact on healthcare in Khartoum State, Sudan	Shortage of pharmacy staff, lack of support from health authorities, lack of training and educational program, lack of job descriptions, lack of specific area in patient files for clinical pharmacist intervention, and low salaries
AbuRuzet al., 2012 [30]	Jordan	Community	Questionnaire based survey	Pharmacists	To study about the role of community pharmacists in Jordan	Lack of pharmaceutical care training, lack of access to medical files, lack of space, physicians’ acceptance, communication with physicians and lack of local data
El Hajj et al., 2016 [32]	Qatar	All areas	Cross-sectional survey	Pharmacists	To examine the extent of pharmaceutical care practice and the barriers to pharmaceutical care provision as perceived by Qatar pharmacists	Inadequate training in pharmaceutical care, lack of documentation skills, lack of pharmaceutical care role models, insufficient opportunity for interaction with other health care providers, inconvenient access to patient medical records, insufficient staff and lack of privacy, time, space
Katoue *et al.,* 2014 [31]	Kuwait	Hospital	Cross-sectional survey	Pharmacists	To investigate hospital pharmacists’ attitudes towards pharmaceutical care, and the barriers to its implementation in Kuwait	Lack of private counselling areas, organizational obstacles, inadequate staff, lack of pharmacist time and adequate technology
Farina et al., 2009 [29]	Brazil	Community	Questionnaire based survey	Pharmacists	To learn about the professional practice of the pharmacists who work in pharmacies and their knowledge and perceptions about pharmaceutical care	Lack of time, of support from the pharmacy owner, and patient’s disinterest

### Pharmacists’ involvement in optimising medicines

**Fig. 1:**
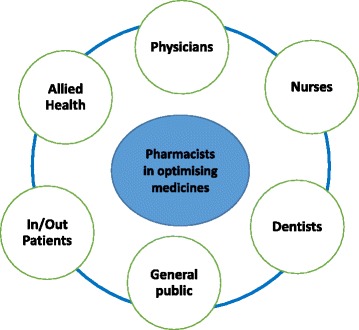
Adequate education and extensive training are important for practicing pharmacists and their pharmaceutical services are central to coordinating and optimising antibiotics among healthcare professionals, patients and the general public

 Practicing pharmacists providing pharmaceutical care services are central to coordinating and optimising medicines among healthcare professionals, patients and the general public is presented in Fig. 1. This centric role of pharmacists is important for the quality use of antibiotics.

### AMR-related interventions led by pharmacists in developing and developed countries

Role of pharmacists’ in enhancing antibiotic use and combating AMR from recently published studies in developing and developed countries are presented in Table 2. The inclusion of pharmacists in different care settings has shown significant improvement in the quality use of antibiotics. Studies showing positive impacts are from both developing [9, 33–35] and developed [10, 36–42] nations. These studies also demonstrate that pharmacists can improve appropriate antibiotic use in hospital [9, 33–37, 39–41] and community [10, 38, 42] settings.

**Table 2:** Success stories of pharmacist intervention around the world in combating AMR from recently published articles

Authors	Country	Setting	Objective	Study design	Type of intervention	Relevant outcomes
Brink *et al.,* 2017 [97]	South Africa	Hospital	To implement an improvement model for existing resources, in order to achieve a reduction in surgical site infections (SSIs) across a heterogeneous group of 34 urban and rural South African hospitals	A pharmacist-driven, prospective audit and feedback strategy	Pharmacist included in the post-pharmacist intervention	pharmacists can effectively improve guideline compliance and sustainable patient outcomes(*P* < 0.0001)
Ellis *et al.,* 2016 [36]	USA	Hospital	To assess the impact of pharmacist intervention on appropriateness of antimicrobial prescribing on a geriatric unit	Pre and Post - pharmacist intervention	Pharmacist included in the post-pharmacist intervention	Post-intervention group had significantly less inappropriate doses for indication compared to the pre-intervention group (*p* = 0.02), pharmacist intervention group had less antibiotics prescribed for an inappropriate duration(*p* < 0.01), post intervention group had medications prescribed with appropriate dose, duration, and indication(*p* = 0.04)
Okada *et al.,* 2016 [37]	Japan	Hospital	To investigate the clinical effectiveness of the pharmacist interventions on antibiotic use	Retrospective study design	Pharmacist included in the intervention group	Effective drug concentrations significantly increased in the intervention group. Intervention (74%) and control (55%).
Northey *et al.,* 2015 [38]	Australia	Community	To assess the effectiveness of involving community pharmacy staff in patient education about antibiotic resistance	Randomized control study	Those in the intervention group were provided with verbal education by pharmacists	Antibiotic knowledge increased after receiving verbal antibiotic education (*p* = 0.008)
Zhou *et al.,* 2015 [34]	China	Hospital	To describe the impacts of pharmacist intervention on the use of antibiotics, particularly in urology clean operations	Pre and Post - pharmacist intervention	Pharmacist included in the post intervention group	Average antibiotic cost decreased, cost of antibiotics as a percentage of total drug cost decreased by 27. 7%; the rate of use of antibiotics decreased from 100% to 7.3%
Booth *et al.,* 2013 [10]	UK	Community	To compare the care pathway of patients with UTI symptoms attending GP services with those receiving management, including trimethoprim supply under PGD, via community pharmacies	Prospective, cross-sectional, mixed methods	Pharmacies invited a purposive sample of female patients to participate	Antibiotic treatments for UTIs could be provided via community pharmacy to improve patient access to treatment which may also maintain antibiotic stewardship and reduce GP workload
Magedanz et al., 2012 [9]	Brazil	Hospital	To assess the impact of ASP, with and without the presence of a pharmacist, in a cardiology hospital in Brazil	Pre and Post pharmacist intervention	Pharmacist included in the post-pharmacist intervention	Adherence to recommendations was increased (64%), hospital antibiotic cost reduction (69%).
Yen *et al.,* 2012 [39]	Taiwan	Hospital	A pharmacist-managed antibiotic intravenous to oral (i.v.-to-p.o.) conversion program has been incorporated to minimize unnecessary i.v. antibiotic usage	Retrospectively collected by chart review	Pharmacist included in the intervention group	Length of hospital stay was significantly decreased (*p* = 0.001)
Dunn *et al*., 2011 [40]	Ireland	Hospital	To assess the impact of the introduction of guidelines and criteria for switching to oral antimicrobials	prospective and of controlled before and after design	Pharmacist included in the intervention group	Duration of IV antimicrobial treatment reduced significantly in the study group post intervention, (*P* = 0.02) compared to the control group
Grill *et al.,* 2011 [41]	Germany	Hospital	To assess the impact of pharmaceutical consulting on the quality of antimicrobial use in a surgical hospital department	Prospective controlled intervention study	Pharmacist included in the intervention group	Intervention reduced the length of antimicrobial courses (IG = 10 days, CG = 11 days, incidence rate ratio for i.v. versus o.p. = 0.88, 95% confidence interval 0.84 to 0.93) and shortened i.v. administration (IG = 8 days, CG = 10 days, hazard rate = 1.76 in favour of switch from i.v. to p.o., 95% confidence interval 1.23 to 2.52). 95% confidence interval 1.23 to 2.52
Shen *et al.,* 2011 [35]	China	Hospital	To evaluate the impact of pharmacist interventions on antibiotic use in inpatients with respiratory tract infections in a tertiary hospital in China	Randomized control study	Pharmacist included in the intervention group, no pharmacist in the control group	Total costs of hospitalization in the intervention group was lower compared to the control group *P* < 0.001. Total cost of antibiotics in the intervention group was lower to the control group (*P* = 0.01). Patients required shorter length of hospital stay (*P* = 0.03)
Hersberger *et al.,* 2009 [42]	Switzerland	Community	To examine prescribing patterns of antibiotics and symptomatic medications for ARTI in Swiss primary care and to monitor pharmacists’ interventions during the prescription-dispensing process	Cluster randomized trial	Pharmacist included in the intervention group	Most patients (80%) were treated only with symptomatic medications. Most frequently prescribed symptomatic ARTI medications were nasal decongestants (39%), cough suppressants (36%), and mucolytic (31%)

*ASP*: Antimicrobial Stewardship Program, *UTI* Urinary Tract Infection, *ARTI* Acute Respiratory Tract Infection, *IV* Intravenous

*GP*: General Physician, *PGD*: Prescribing guidelines

## Discussion

### Statement of principal findings

Significant progress in delivering pharmaceutical care services by the pharmacists has occurred within the pharmacy profession in the past few decades. There is a notable shift from medicine supply roles to pharmacist-delivered patient-centred services for the purpose of improving rational use of medications and ultimately enhancing the quality of life of patients. In many developed nations, pharmaceutical care services as a practice based profession was introduced many years ago. These developed nations are now experiencing the positive outcomes of the expanded roles of pharmacists, which directly impact patient care and quality use of medicines. However, in many developing countries, pharmaceutical care services are still limited to traditional pharmacy practices such as procurement of drugs, extemporaneous compounding, dispensing of prescriptions and selling of medicines. Therefore, an understanding the expanded roles of pharmacists in the current rapidly changing healthcare system of developed countries and the benefits they can bring is important for the development of healthcare systems in developing countries. One of the major outcomes of enhanced pharmacists’ roles is the more appropriate use of antibiotics among health care professionals, patients in different settings of care and the general public. The importance of pharmacist-led antibiotic stewardship programs has long been recognised in the developed nations. The establishment of well qualified and trained pharmacy workforce in developing countries has the potential to reduce antibiotics overuse and misuse.

### Context of these findings

#### Pharmacists’ role in developing countries

The pharmacy profession still has an emerging professional status in developing countries [43]. Pharmacy services in developing countries tend to be limited to roles in drug manufacturing, medicines procurement, drug dispensing and drug supply chain and storage [44]. Pharmacists are well recognized and well remunerated for their role within the pharmaceutical industry in developing countries [43] and there is a strong demand for employment in this field. However, in developing countries, pharmaceutical care services, clinical roles or pharmacy practice more generally are not yet well recognised as key roles for pharmacists [23]. In contrast, in developed nations pharmaceutical care services are considered as integral components of the health care systems [45]. Pharmacists in developing countries are not actively involved in providing patient care related services [46] such as infection control, immunization, diabetes, lipid management, coronary heart disease, skin cancer prevention, mental health, sexual health, prevention of substance abuse, smoking cessation, nutrition and physical activity [45, 47] and there is limited professional involvement in initiatives related to rational medicine use [47]. Pharmacists are underutilized, their education and training under-recognised and their potential roles in patient care can be viewed as missed opportunities for optimising the health of populations in developing countries [48, 49].

There are numerous challenges and barriers faced by pharmacists in developing countries to implement and maintain sustainable pharmaceutical care services [23, 27, 30]. Most developing countries are struggling with a shortage of pharmacists [27, 28, 31, 32, 43], inadequate education and training for pharmacists [24, 25, 27, 28, 30, 32], and support from other health care professionals [23–25, 28, 30, 32]. Even though pharmacists from developing countries show great enthusiasm to promote and to devote themselves to the provision of pharmaceutical care services in their countries [27], efforts made to institutionalize pharmaceutical care services is minimal in many developing countries [27, 45, 47]. The underdeveloped nature of pharmaceutical care services in developing countries is considered a primary reason for inappropriate and inadequate medication practices [45, 47].

If pharmacists are to provide patient care services in developing countries, it is essential to remove these major barriers [50]. These could be overcome by increasing employment opportunities for pharmacists, improving educational programmes and facilitating effective collaboration with other health professionals [46]. It is essential that adequate education and extensive training of pharmacists is provided by the tertiary education sector of developing countries, and that integrate pharmacists’ roles are integrated with those of physicians and other health care providers in the health care systems of developing countries to optimise patient care, and ultimately reduce health care costs and improve public health.

#### Pharmacists’ contributions in developed nations

Extended pharmacists’ roles are well established in the developed nations [51]. Pharmacist-delivered pharmaceutical care services have long been established and are widely developed in the United States (US) [52], United Kingdom (UK) [10], Canada [53], Australia [54, 55] and New Zealand [56]. They have produced notably positive outcomes in both hospital [52] and community settings [10] in these countries. Adequate education and extensive training of pharmacists in these countries has produced pharmacists, able to deliver high quality pharmaceutical care services [57]. As a result, these countries have integrated pharmacists as an important members of their health care teams [58]. Acceptance of pharmacists’ extended roles by physicians and other health care workers has produced significant positive outcomes in patient care in these countries [59, 60]. The numerous interventions in medication management reported in these countries demonstrate the high level of trust of pharmacists by the general public in these countries [61].

An example of an expanded pharmacist roles includes clinical pharmacist activities in Australian hospital emergency departments (ED) [62]. ED medical and nursing staff highly value pharmacist-delivered clinically significant medication interventions at an early stage of patient entry into the hospital. Similarly, the important role of the community pharmacy in the health sector is well established in many developed nations [10] with, for example, pharmacists interventions leading to reduced prescription errors in the community setting [63].

In developed nations, pharmacists’ role has expanded to provide a variety of services including pharmacovigilance activities [64], pharmacist prescribing [65], disease state management [66], medication reconciliation– inpatient [67] and outpatient [68], discharge management and counselling [69], home and residential aged care medication reviews [70], pharmacists in general practices [71], and pharmacists-led clinics (diabetes and cardiovascular) [72]. The provision of and outcomes from these expanded services are appreciated by physicians [73, 74] and the public [75, 76]. Such enhanced pharmacist roles in developed nations have demonstrated improved clinical and therapeutic outcomes [77] and reduced health care costs [53]. The expanded roles require appropriate education and training programs for pharmacists and integration of pharmacists into the health care system. Consequently it is recommended that developing countries through policy and program development and adoption ensure appropriate education and training programs for pharmacists and implement healthcare practice frameworks that recognise the potential of such training and education and integrate pharmacists into their healthcare systems and multidisciplinary delivery of patient care.

#### Pharmacists’ contributions in combating AMR in both developed and developing nations

Pharmacists’ contribution in optimal antimicrobial use is an essential component in the fight against growing AMR. Many developed countries have achieved success with the implementation of antimicrobial stewardship (AMS) programs that include pharmacists. [78, 79] AMS programs aim to ensure judicious, appropriate and safe antibiotic use. Guntenet al*.,* 2007 [80], clearly describes the clinical and economic outcomes that can result from antibiotic-related pharmaceutical services. In this review, pharmacist’s successful contributions to reducing the development and spread of AMR have been identified. The crucial role of pharmacists in promoting safe and cost-effective use of antimicrobial agents is acknowledged and implemented in the healthcare systems of numerous countries [9, 10, 33–42]. A noteworthy example of this role is their role in the care of critically ill patients with infectious diseases in Intensive Care Units (ICU) which results in improved clinical and economic outcomes [81].

In contrast, developing countries have not yet implemented pharmacist-led initiatives such as pharmacist-led AMS programs [82]. In these countries, pharmacists could play a key role in minimizing unnecessary prescribing of antibiotics and developing local prescribing guidelines according to diagnoses and local antibiotic susceptibility patterns [83]. Education regarding infection-control practices is also an important avenue for pharmacist involvement. Given their ready accessibility by the public, community pharmacists should be proactive in regard to educating the public about important infection-control practices such as general hygiene, hand hygiene, cough etiquette, immunizations and staying home when sick. Although these strategies may seem like common sense, patient understanding of these basic infection-control practices should not be overestimated. Adequately trained pharmacists can educate patients about management of viral, bacterial and common fungal infections.

Hence, there is a necessity to ensure suitably qualified pharmacists in developing countries through delivery of high quality and expanded pharmacy education. Education on infectious diseases, their management and AMR should be given increased attention in the pharmacy curriculum. The resultant knowledge, behaviours and skills should be evaluated prior to qualification and continuing professional development in this therapeutic area maintained if developing countries are to avoid the negative impact of inappropriate antibiotic utilization and AMR that is forecast in the developing countries in the near future [84].

### Strengths and weaknesses of the study

To our knowledge, there have been few reviews of the actual or potential role of pharmacists in developing countries that combat the challenge of AMR. This review explored the current situation of pharmacists in developing countries, the role of pharmacists in pharmaceutical care in the developed world and the success stories of pharmacist-related interventions in advancing prudent antibiotic use. This review highlights significant justification for enhancing pharmacists’ roles in developing countries and the steps that should be taken by these countries to achieve these expanded pharmacists’ roles. This review was limited to studies published in English, and therefore relevant studies published non-English languages would have been missed.

### Meaning of this review: Possible mechanisms and implications for clinicians or policymakers

Given the emerging nature of pharmaceutical care services, some developing countries have incorporated pharmaceutical care courses, such as clinical pharmacy, albeit in their infancy, within the national pharmacy curriculum [85]. However, many developing countries remain reluctant to offer a clinically significant role for pharmacists within their healthcare systems. For example, there are very few defined positions for clinical pharmacists in hospitals and within the health care sector in many developing countries [86]. Adequate hospital access for clinical training of pharmacy students remains a barrier [87]. Finding well qualified and experienced professionals to lead teaching in clinical pharmacy, is a further challenge. Some countries have managed to recruit experienced international clinical pharmacy educators to develop and support clinical pharmacy education programs that include pharmacist-led models of care [88, 89]. If developing countries are to avoid the consequences of indiscriminate and inappropriate antibiotic use it is essential that the knowledge and skills of all members of the healthcare team be harnessed including those of pharmacists and that the opportunity to deliver this knowledge and skills is incorporated within the healthcare system. Pharmacists with appropriate educational background and clinical training can play a crucial role in reducing the burden of antimicrobial resistance in hospital settings [90]. Interventions minimizing inappropriate use of antibiotics by clinical pharmacists are well reported in literature [91–93]. Pharmacists can assist to eliminate the misconceptions about antibiotic use and educate the patients, prescribers and other health care professional on appropriate antibiotic utilization.

Furthermore, pharmacists can provide their services and support to physicians, nurses and other healthcare professionals to optimize antibiotic use, provide updated information about antibiotics and the adverse drug effects of antibiotics and monitor antibiotic use within healthcare settings. Establishing collaborative team based relationships between physicians and pharmacists will allow for improved patient outcomes [94]. Antibiotic guidelines are not available in many developing countries, and pharmacists can play a key role in their development [95]. Counselling of patients by pharmacists about appropriate antibiotic use is an important role for pharmacists [96] and would be facilitated by the implementation of pharmaceutical service provision in developing countries. [70].

### Unanswered questions and future research

While the evidence for expanded pharmacist roles in developed countries is robust and generally translatable, it is nevertheless important that local evaluation of interventions by appropriately educated and trained pharmacists occurs. Factors related to success and failure of local programs will need to be identified to inform further program development and implementation. Importantly, programs expanding clinical pharmacist services are likely to be assisted with local and national policy and appropriate remuneration. Initiatives such as guideline development will also assist these programs. Although this review has focussed on pharmacist education and training, the training of other healthcare professionals also requires evaluation and possible overhaul. Education that highlights the benefits of health care provision when providers are team players will be advantageous.

## Conclusion

As has occurred in developed countries, the provision of qualified trained pharmacists and pharmacist integration into healthcare systems has the potential to make a significant impact on inappropriate antibiotic use in developing countries. Although this is only one of a number of interventions necessary to overcome the problem of catastrophic AMR in developing countries, it is achievable. Studies from developed countries have demonstrated that cost-effective optimal patient outcomes are achieved when pharmacists roles are enhanced within the health care system, when they are recognised as part of the health care team to provide advice on the rational and safe use of medicines, and university curricula and continuing professional education provide theoretical and practical knowledge and skills regarding the quality use of medicines. Antimicrobial stewardship involving pharmacists should be established in hospitals to ensure judicious and appropriate antimicrobial use. Adoption of these strategies will assist developing countries overcome their increased vulnerability to growing AMR.
